# Obesity, Mortality, and Life Years Lost Associated With Breast Cancer in Nonsmoking US Women, National Health Interview Survey, 1997–2000

**DOI:** 10.5888/pcd10.130112

**Published:** 2013-11-14

**Authors:** Su-Hsin Chang, Lisa M. Pollack, Graham A. Colditz

**Affiliations:** Author Affiliations: Lisa M. Pollack, George Warren Brown School of Social Work, Washington University in St Louis, St Louis, Missouri; Graham A. Colditz, Washington University School of Medicine, St Louis, Missouri.

## Abstract

**Introduction:**

The relationship between obesity and breast cancer has been extensively investigated. However, how obesity and breast cancer interplay to affect mortality and life expectancy of women in the United States has not been well studied.

**Methods:**

We used data from the National Health Interview Survey, 1997–2000. Our sample included nonsmoking, nonpregnant women who reported a body mass index of at least 18.5 kg/m^2^ and no cancer other than breast cancer at the time of the survey. A survival model with Gamma frailty and Gompertz baseline was used to estimate relative risks of total mortality and project life years lost associated with breast cancer by obesity status and age.

**Results:**

Breast cancer increased risk of mortality depending on degree of obesity and decreased life years by 1 to 12 years depending on race, age, and obesity status. Relative risks for death increased with degree of obesity. Obese women under age 50 across all racial groups were predicted to lose the most life years; racial groups other than whites and blacks lost the most life years (11.9 y), followed by whites (9.8 y) and blacks (9.2 y).

**Conclusion:**

The number of life years lost associated with breast cancer was more marked for more obese than for less obese women and for women under age 50 and women aged 70 or older than for women aged 50 through 69. Public health initiatives should put more emphasis on the prevention and control of obesity for these target populations.

## MEDSCAPE CME

Medscape, LLC is pleased to provide online continuing medical education (CME) for this journal article, allowing clinicians the opportunity to earn CME credit.

This activity has been planned and implemented in accordance with the Essential Areas and policies of the Accreditation Council for Continuing Medical Education through the joint sponsorship of Medscape, LLC and Preventing Chronic Disease. Medscape, LLC is accredited by the ACCME to provide continuing medical education for physicians.

Medscape, LLC designates this Journal-based CME activity for a maximum of 1 **AMA PRA Category 1 Credit(s)™**. Physicians should claim only the credit commensurate with the extent of their participation in the activity.

All other clinicians completing this activity will be issued a certificate of participation. To participate in this journal CME activity: (1) review the learning objectives and author disclosures; (2) study the education content; (3) take the post-test with a 70% minimum passing score and complete the evaluation at www.medscape.org/journal/pcd (4) view/print certificate.

**Release date: November 13, 2013; Expiration date: November 13, 2014**

### Learning Objectives

Upon completion of this activity, participants will be able to:

- Analyze the effect of overweight and obesity on the risk of mortality among women with breast cancer
- Compare the effects of obesity on the risk of mortality among women with and without breast cancer
- Assess the effect of age on the relationship between obesity and the risk of mortality among women with breast cancer
- Assess the effect of race/ethnicity on the relationship between obesity and the risk of mortality among women with breast cancer

**EDITORS**

Teresa Ramsey, Editor, *Preventing Chronic Disease*. Disclosure: Teresa Ramsey has disclosed no relevant financial relationships.

**CME AUTHOR**

Charles P. Vega, MD, Associate Professor and Residency Director, Department of Family Medicine, University of California, Irvine. Disclosure: Charles P. Vega, MD, has disclosed no relevant financial relationships.

**AUTHORS AND CREDENTIALS**

Disclosures: Su-Hsin Chang, Lisa M. Pollack, and Graham A. Colditz have disclosed no relevant financial relationships.

Affiliations: Su-Hsin Chang, PhD, Division of Public Health Sciences, Department of Surgery, Washington University School of Medicine, St. Louis, Missouri; Lisa M. Pollack, MPT, MPH, George Warren Brown School of Social Work, Washington University, St. Louis, Missouri; and Graham A. Colditz, MD, DrPH, Washington University School Of Medicine, St. Louis, Missouri.

## Introduction

Breast cancer is the most commonly diagnosed cancer in women and the second leading cause of death among women (1). Obesity is a known risk factor for postmenopausal breast cancer (2,3) but may be inversely related to premenopausal breast cancer incidence (4). Obesity is also associated with increased all-cause mortality (5–7). However, it is unclear how obesity and breast cancer interplay to affect mortality and life expectancy of adult women. Some studies have focused on obesity in relation to breast cancer incidence (8–10); others have explored the relationship between obesity and mortality from breast cancer (11–13). Few studies have examined life expectancy associated with breast cancer (14,15). To our knowledge, no studies have considered this association in relation to body mass index (BMI). Our study is the first to consider BMI in the relationship between breast cancer and mortality rate as well as life years lost associated with breast cancer. Our study used both life years lost and mortality rate, which are accepted outcome measures of population health (16,17).

The goal of this study is to investigate the mortality risks associated with breast cancer according to BMI level and race and to predict life years lost among women in the United States. We used data from a national probability sample of the US population and a class of survival model. The projection of life years is computed on the basis of mortality estimates and the observed characteristics of the sample as a snapshot of their life span. This approach is not restricted by the length of mortality follow-up and the observed mortality rates, and thus provides an alternative to the life table approach (14).

## Methods

### Data and the sample

Data from the National Health Interview Survey (NHIS) and the NHIS Linked Mortality Public-Use Files were used in this study (18). The NHIS is a cross-sectional household interview survey providing health information on the civilian, noninstitutionalized household population of the United States. The NHIS consists of 3 major components: Family, Sample Adult, and Sample Child. From each family, 1 sample adult (aged 18 years or older) and 1 sample child (if any children under age 18 are present) are randomly selected, and information on each is collected from the sample adult core and the sample child core questionnaires. The Sample Adult Data Files from 1997 to 2000 were used and linked to the data in the NHIS Linked Mortality Public-Use Files by personal identification numbers. Mortality data followed up the NHIS sample from the date of interview through December 31, 2006.

We focused on female breast cancer; therefore, our sample included only women. The sample exclusion criteria were the following: 1) women with any missing data; 2) women who had smoked more than 100 cigarettes in their entire life, because analyses can be confounded by illnesses associated with smoking (6,7); 3) women pregnant at the time of survey, because BMI levels are unstable during pregnancy; 4) women who reported having had any type of cancer other than breast cancer, to exclude the possibility of breast cancer as a secondary cancer; and 5) underweight women, because disease-driven weight loss could perplex the analysis.

### Outcome variable and covariates

The outcome variable was age at death or censor (December 31, 2006). We controlled for breast cancer, BMI (self-reported), race, age at the time of survey, alcohol consumption, physical activity, and educational attainment. Breast cancer information included breast cancer status and the duration from the time of diagnosis to that of the survey. For women with no breast cancer diagnosis reported, the duration was recorded as zero. BMI classifications were based on the standards established by the World Health Organization (19): overweight, BMI 25.0–29.9 kg/m^2^; class I obese, BMI 30.0–34.9 kg/m^2^; class II obese, BMI 35.0–39.9 kg/m^2^; and class III obese, BMI 40 kg/m^2^ or higher. Normal-weight women (BMI 18.5–24.9 kg/m^2^) were the reference group. Age at survey was described using 5 age categories: 30 to 39, 40 to 49, 50 to 59, 60 to 69, and 70 years or older. Age 30 or under was the reference category. For race, dichotomized variables for whites and blacks were used. Racial groups other than whites and blacks were the reference group. Interaction terms of breast cancer status with BMI, age, and racial groups were included as covariates.

The additional covariates were dichotomized as follows: educational attainment (whether a person was a high school graduate); alcohol consumption (whether a person had had no more than 12 drinks of any type of alcoholic beverage in the person’s entire life at time of survey); and physical activity (whether a person engaged in light/modest or vigorous physical activity for at least 10 minutes more than once per week).

### Statistical analysis

A survival model with Gamma frailty and Gompertz baseline was used (20). Maximum pseudo-likelihood estimation was performed to adjust for complex sampling design in the NHIS data (21). The estimates were used to compute hazard ratios for death (relative risks, RRs) (22) by BMI and racial groups for people with breast cancer and without any cancer. Life years were predicted by a closed-form expression (23). The sample was divided into subsamples with different combinations of race (whites, blacks, and other), age (aged <50 y, 50–69 y, and ≥70 y), and obesity status (obese: class I–III; nonobese: normal weight and overweight). For each subsample, a sample was selected on the basis of sampling weights, and life years were predicted on the basis of characteristics of the subsample. Within the same subsample, the number of life years lost was projected by comparing the predicted life years of women with breast cancer to the predicted life years of the same women had they not had breast cancer. The bootstrap method was performed to resample the subpopulations 1,000 times to compute the means and standard errors (24).

All estimations and bootstraps were adjusted for the complex sampling design in the NHIS (25). Stata version 11 (Stata Corp LP, College Station, Texas) was used to obtain the summary statistics for the sample and the population, and Matlab version 7.13, R2012b (MathWorks Inc, Natick, Massachusetts) was used to perform estimations and predictions.

## Results

The initial sample size of women in our data was 70,651. The remaining sample after applying the exclusion criteria consisted of 35,853 women, representing a US population estimated at 51,860,143 (95% confidence interval [CI], 50,944,982–52,775,304) nonsmoking adult women (aged ≥18 y) (Figure 1). Of the population, 78% were white and 14% were black (Table 1). The average BMI was 26.3 kg/m^2^ (Table 2); 50% of the population had normal weight; 28% had a BMI within the overweight range; 14% belonged to class I obese; 5% belonged to class II obese; and 3% fell into class III obese (Table 1).

**Table 1:** Descriptive Statistics for Nonsmoking US Women in the Sample and the Population From the National Health Interview Survey, 1997–2000^a^

Variable	Breast Cancer		No Cancer		Total	
	n	N (SE)	n	N (SE)	n	N (SE)
**Total**	771	1,058,767 (43,768)	35,082	50,801,376 (459,460)	35,853	51,860,143 (465,260)
**All-cause death**	208	260,108 (20,253)	2,438	2,844,044 (76,011)	2,646	3,104,152 (795,920)
**Race**						
White	661	924,138 (41,498)	25,941	39,459,691 (389,888)	26,602	40,383,829 (394,164)
Black	76	84,151 (9,755)	6,060	7,219,083 (202,540)	6,136	7,303,234 (205,566)
Other	34	50,478 (109,29)	3,081	4,122,602 (128,190)	3,115	41,73,080 (128,575)
**BMI group^b^**						
Normal weight	315	433,178 (26,915)	16,859	25,531,527 (289,971)	17,174	25,964,705 (291,154)
Overweight	269	379,179 (28,156)	10,235	14,356,072 (196,833)	10,504	14,735,251 (201,082)
Class I obese	136	175,026 (16,906)	5,043	6,881,706 (124,430)	5,179	7,056,731 (125,298)
Class II obese	34	53,465 (9,871)	1,800	2,471,069 (73,607)	1,834	2,524,534 (73,495)
Class III obese	17	17,920 (4,738)	1,145	1,561,003 (57,872)	1,162	1,578,922 (58,002)
**Age group, y**						
<30	7	11,715 (5,337)	7,664	12,188,840 (207,881)	7,671	12,200,555 (208,345)
30–39	19	26,668 (6,681)	7,850	10,964,477 (160,630)	7,869	10,991,145 (160,655)
40–49	65	100,383 (14,445)	6,437	10,061,258 (176,813)	6,502	10,161,641 (177,674)
50–59	113	168,871 (19,351)	4,248	6,461,688 (122,378)	4,361	6,630,559 (123,705)
60–69	153	221,380 (18,495)	3,342	4,567,730 (99,367)	3,495	4,789,110 (103,801)
≥70	414	529,750 (28,844)	5,541	6,557,384 (129,219)	5,955	7,087,134 (136,195)
**Education^c^**						
≥ High school graduate	585	821,615 (38,200)	27,156	41,660,054 (412,085)	27,741	42,481,669 (417,161)
< High school graduate	186	237,152 (20,158)	7,926	9,141,323 (174,170)	8,112	9,378,475 (174,589)
**Alcohol use^d^**						
≤12 drinks	143	190,923 (17,843)	4,245	5,928,126 (118,714)	4,388	6,119,050 (119,701)
>12 drinks	628	867,844 (40,341)	30,837	44,873,250 (427,473)	31,465	45,741,094 (433,428)
**Physical activity^e^**						
Yes	303	451,699 (29,693)	16,760	26,152,738 (318,522)	17,063	26,604,438 (321,812)
No	468	607,068 (31,665)	18,322	24,648,638 (277,708)	18,790	25,255,706 (279,927)

Abbreviations: BMI: body mass index; SE: standard error.

a Data sources: National Health Interview Survey (NHIS), 1997–2000 and the NHIS Linked Mortality Public-Use Files.

b Normal weight: BMI 18.5–24.9 kg/m^2^; overweight: BMI 25.0–29.9 kg/m^2^; class I obese: BMI 30.0–34.9 kg/m^2^; class II obese: BMI 35.0–39.9 kg/m^2^; class III obese: BMI ≥40 kg/m^2^.

c Whether an individual was a high school graduate.

d Whether a person had had no more than 12 drinks of any type of alcoholic beverage in the person’s entire life at time of survey.

e Whether an individual engaged in light/modest or vigorous physical activity for at least 10 min more than once per week.

**Table 2:** Continuous Variables for Nonsmoking US Women From the National Health Interview Survey, 1997–2000

Variable	Breast Cancer		No Cancer		Total	
	Sample Mean	Population Mean (SE)	Sample Mean	Population Mean (SE)	Sample Mean	Population Mean (SE)
**BMI, kg/m^2^**	26.9	26.8 (0.18)	26.6	26.3 (0.04)	26.6	26.3 (0.04)
**Duration from breast cancer diagnosis to survey, y**	7.7	9.8 (0.42)	0.0	0.0	7.7	9.8 (0.42)
**Mortality follow-up, y**	6.7	8.5 (0.04)	7.3	8.5 (0.01)	7.3	8.5 (0.01)
**Age at survey, y**	67.8	66.8 (0.59)	46.8	44.6 (0.15)	47.3	45.0 (0.16)
**Age at death, y**	79.5	78.4 (0.97)	79.1	77.9 (0.35)	79.1	77.9 (0.33)

Abbreviations: BMI, body mass index; SE, standard error.

**Figure:**
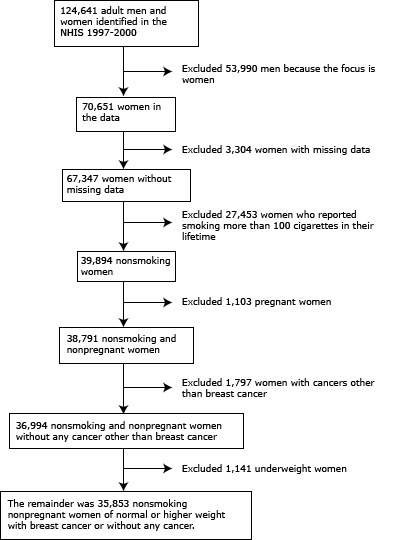
Data attrition diagram, study on obesity, mortality, and life years lost associated with breast cancer in nonsmoking US women, National Health Interview Survey (NHIS), 1997–2000.

A total of 771 women in the sample (representing an estimated population of 1,058,767 [95% CI, 972,625–1,144,909]) reported having had a breast cancer diagnosis before the survey. The mean time from their cancer diagnosis to the time of survey was 9.8 years (95% CI, 9.0–10.6) for the population (Table 2). The mean age of the population was 45 years (95% CI, 44.7–45.3). Breast cancer survivors were older on average (66.8 y; 95% CI, 65.6–67.9) than women who did not report any cancer (44.6 y; 95% CI, 44.3–44.9). By 2006, a total of 3,104,152 (95% CI, 2,947,591–3,260,714) all-cause deaths were estimated in this population (260,108 deaths for breast cancer survivors and 2,844,044 deaths for women without any cancer). The mean age at death was estimated at 78.4 (95% CI, 76.5–80.4) for breast cancer patients and 77.9 (95% CI, 77.2–78.6) for women without any cancer in the population.

We analyzed the adjusted relative risks of total mortality for women with breast cancer and without any cancer at survey by BMI group. In the presence of breast cancer, relative risks increased with degree of obesity: 1.63 (95% CI, 1.28–2.07) for the class I obese, 2.66 (95% CI, 2.16–3.29) for the class II obese, and 8.49 (95% CI, 6.20–11.64) for the class III obese. However, overweight women did not appear to be at an increased risk of mortality (RR, 0.90; 95% CI, 0.71–1.13). In the absence of any cancer, any degree of obesity increased mortality risk: 1.10 (95% CI, 1.07–1.13) for overweight, 1.18 (95% CI, 1.14–1.21) for the class I obese, 1.90 (95% CI, 1.81–1.99) for the class II obese, and 2.88 (95% CI, 2.72–3.06) for the class III obese.

The risk of mortality was higher for black women (RR, 1.29; 95% CI, 1.21–1.36) than for white women (RR, 0.88; 95% CI, 0.83–0.93) when cancer was not reported. When breast cancer was reported, relative risks were 1.03 (95% CI, 0.82–1.30) for whites and 1.59 (95% CI, 1.29–1.95) for blacks, compared with racial groups other than blacks and whites. It should be noted that some regression coefficients were not significant, possibly because of multicollinearity resulting from adding interaction terms (26). However, the overall model fit was significant (*F* = 117.11, *P* value < .001).

Across all ages, obese (class I–III obese) women with breast cancer lost more life years than their nonobese (normal-weight and overweight) counterparts. Obese whites lost 4.7 (95% CI, 4.1–5.3) life years; obese blacks lost 4.7 (95% CI, 3.0–6.4) years; and obese racial groups other than whites and blacks lost approximately 5.1 (95% CI, 2.9–7.4) life years. Nonobese breast cancer patients lost fewer life years than their obese counterparts: 3.5 (95% CI, 3.1–3.9) years for whites, 3.5 (95% CI, 2.2–4.8) years for blacks, and 4.0 (95% CI, 2.6–5.4) years for all other racial groups.

If breast cancer was present in obese women aged 50 or less, life years lost were 9.8 years (95% CI, 7.6–12.0) for whites, 9.2 (95% CI, 6.4–12.0) for blacks, and 11.9 (95% CI, 5.7–18.0) for all other racial groups. Fewer life years were lost for nonobese women aged 50 or less: 7.9 years (95% CI, 6.7–9.0) for whites, 5.3 (95% CI, 3.0–7.7) for blacks, and 6.0 (95% CI, 5.7–6.2) years for all other racial groups (Table 3). Obese women aged 50 through 69 lost more life years associated with breast cancer than nonobese women of the same age: 3.8 years (95% CI, 3.0–4.7) for obese whites, compared with 1.3 (95% CI, 0.6–2.0) for nonobese whites; 2.5 (95% CI, 0.97–4.1) for obese blacks, compared with 0.6 (95% CI, −1.4−2.6) for nonobese blacks; and 3.8 (95% CI, 2.3−5.2) for obese members of other racial groups compared with 1.7 (95% CI, −0.2−3.7) for nonobese racial groups other than whites and blacks.

**Table 3:** Predicted Life Years Lost Associated With Breast Cancer for Nonsmoking US Women by Obesity Status, Age, and Race, National Health Interview Survey, 1997–2000 **a**

Obesity Status	Age, y	White, y (SE)	Black, y (SE)	Other, y (SE)
Obese	All	4.66 (0.31)	4.69 (0.85)	5.12 (1.16)
	<50	9.77 (1.13)	9.22 (1.42)	11.85 (3.12)
	50–69	3.84 (0.41)	2.52 (0.79)	3.75 (0.72)
	≥70	4.93 (0.51)	6.87 (0.15)	5.17 (0)
Nonobese	All	3.53 (0.21)	3.48 (0.65)	3.97 (0.71)
	<50	7.85 (0.61)	5.32 (1.22)	5.97 (0.13)
	50–69	1.31 (0.35)	0.62 (1.02)	1.74 (0.99)
	≥70	3.68 (0.25)	4.48 (0.78)	5.81 (0.62)

Abbreviations: SE: standard error.

a Data sources: National Health Interview Survey (NHIS), 1997–2000 and the NHIS Linked Mortality Public-Use Data Files.

A similar trend was found among women aged 70 or older. For obese women aged 70 or older, the number of life years lost was 4.9 (95% CI, 3.9−5.9) for whites and 6.9 (95% CI, 6.6−7.2) for blacks. With the exception of racial groups other than whites and blacks, the number of life years lost was less for nonobese women aged 70 years or older: 3.7 (95% CI, 3.2−4.2) for whites, 4.5 (95% CI, 3.0−6.0) for blacks, and 5.8 (95% CI, 5.0−7.0) for all other racial groups.

## Discussion

We used the following examples to translate life years lost associated with breast cancer to the proportion of total life years. An obese white woman under age 50 with breast cancer was expected to lose 9.8 years of life, which translates to a 12.1% reduction in the expected total life years (81.4 years) for a white woman aged 40 years (27). Similarly, an obese black woman under age 50 with breast cancer was expected to lose 9.2 years of life, which translates to an 11.9% reduction of the total life years (77.5 years) for a black woman aged 40 years (27).

No obvious racial disparity was found in life years lost associated with breast cancer between blacks and whites. Whites (obese and nonobese) lost more life years than their black counterparts for age groups of under 50 and 50 to 69, whereas black women aged 70 years or older lost more life years than white women of the same age range. A plausible explanation is that our data showed fewer breast cancer cases in blacks than in whites and, on average, black women have a higher prevalence of obesity and a shorter life expectancy than white women (1). Therefore, the extent to which breast cancer shortened life years was not as prominent as the extent to which breast cancer increased mortality risk in blacks. Wong et al (28) used a stochastic model to simulate life expectancy for blacks and whites. They found that, of all cancers, breast cancer had the biggest impact on the racial difference in life years lost, accounting for 0.15 years (life years lost for blacks minus that for whites), compared with 0.03 years for obese women and −0.05 years for nonobese women at all ages in our study. The smaller gap between blacks and whites in our study was because the differences were positive and wider for women aged 70 or older (1.94 years for obese women and 0.80 years for nonobese women), whereas the differences were negative for women aged less than 70.

Previous studies that analyzed breast cancer mortality and BMI found results comparable to those of our study: higher mortality among breast cancer patients with a higher degree of obesity. Reeves et al analyzed 1.2 million UK women aged 50 to 64 from 1996 to 2001 and examined mortality from breast cancer in relation to BMI (10). Like us, they found that increasing BMI was associated with a significant increase in the risk of breast cancer mortality. By using data from 1997, Ni Mhurchu et al examined postmenopausal breast cancer mortality in New Zealand and found that higher than optimal BMI (>21 kg/m^2^) contributed to postmenopausal breast cancer deaths (29); high BMI contributed to 4% of cancer deaths, though the proportion of these deaths that was specific to breast cancer is unknown (29). Our results with regard to mortality rate are consistent with those of Aragón et al, who found that premature mortality attributable to breast cancer among San Francisco residents was the highest among black women compared with other ethnic groups (Asians/Pacific Islanders, Latinos/Hispanics, and whites) (14). However, the predicted life years lost in their study (13.4 years) was generally higher than that in ours, because we focused on nonsmoking women with no other cancer reported at survey and controlled for several confounding variables.

Our study has several strengths. First, our analysis focused on breast cancer and examined its association with mortality and life years, which has not been well studied and deserves more investigation (14,15). Second, the major outcomes of our study are the basic metrics for population health and provide information about mortality and longevity (16). Third, to our knowledge, ours is the first study to incorporate BMI, age, and race information in the analysis of breast cancer, mortality, and life years lost. Fourth, our study advances methodology by controlling for unobserved heterogeneity, allowing for a more precise estimation and prediction of life years lost. This is important for population studies, which lack information specific to breast cancer. Last, we used a national data set, and our conclusions are generalizable to the population of US women.

This study has several limitations, many of which are due to secondary data constraints. First, breast cancer status was dichotomized, preventing us from differentiating the extent of the disease progression or the effects of different types of breast cancers. Second, the data contained few breast cancer cases for women aged less than 40 and for women other than whites and blacks. The predicted life years lost for adult women under 50 and for racial groups other than whites and blacks should be interpreted with this caveat in mind. Third, BMI information was self-reported and recorded only at the time of survey, the latter of which restricted analyses of BMI and mortality at the diagnosis of breast cancer. Fourth, the data used were cross-sectional, and our model is not time-varying and does not capture the dynamics of disease evolution or weight change over time, limiting its projection capability on life years (30,31). Last, our data does not capture menopausal status — shown to differentiate the associations of BMI with breast cancer mortality risk (10) — but this limitation was compensated for by dividing the sample into 3 age groups (<50 y, 50−69 y, and ≥70 y). Future studies should focus on how the extent of breast cancer progression, different types of breast cancers, treatments, or menopausal status affect the prediction of life years lost associated with breast cancer.

Our study suggests that breast cancer increased the risk of early mortality depending on degree of obesity and decreased life years by 1 to 12 years depending on race, age, and obesity status. The number of life years lost associated with breast cancer was higher for more obese women aged less than 50 than for nonobese women of the same age. Moreover, obese women with breast cancer aged less than 50 and older than 70 bear a higher burden in terms of years of life lost. Public health initiatives should put more emphasis on the prevention and control of obesity for these target populations.

## Figures and Tables

**Table:** 

**1. The activity supported the learning objectives.**				
**Strongly Disagree**				**Strongly Agree**
1	2	3	4	5
**2. The material was organized clearly for learning to occur.**				
**Strongly Disagree**				**Strongly Agree**
1	2	3	4	5
**3. The content learned from this activity will impact my practice.**				
**Strongly Disagree**				**Strongly Agree**
1	2	3	4	5
**4. The activity was presented objectively and free of commercial bias.**				
**Strongly Disagree**				**Strongly Agree**
1	2	3	4	5

## Post-Test Information

To obtain credit, you should first read the journal article. After reading the article, you should be able to answer the following, related, multiple-choice questions. To complete the questions (with a minimum 70% passing score) and earn continuing medical education (CME) credit, please go to http://www.medscape.org/journal/pcd. Credit cannot be obtained for tests completed on paper, although you may use the worksheet below to keep a record of your answers. You must be a registered user on Medscape.org. If you are not registered on Medscape.org, please click on the "Register" link on the right hand side of the website to register. Only one answer is correct for each question. Once you successfully answer all post-test questions you will be able to view and/or print your certificate. For questions regarding the content of this activity, contact the accredited provider, CME@medscape.net. For technical assistance, contact CME@webmd.net. American Medical Association's Physician's Recognition Award (AMA PRA) credits are accepted in the US as evidence of participation in CME activities. For further information on this award, please refer to http://www.ama-assn.org/ama/pub/about-ama/awards/ama-physicians-recognition-award.page. The AMA has determined that physicians not licensed in the US who participate in this CME activity are eligible for **AMA PRA Category 1 Credits™**. Through agreements that the AMA has made with agencies in some countries, AMA PRA credit may be acceptable as evidence of participation in CME activities. If you are not licensed in the US, please complete the questions online, print the AMA PRA CME credit certificate and present it to your national medical association for review.

## Post-Test Questions

### Article Title: Obesity, Mortality, and Life Years Lost Associated With Breast Cancer in Nonsmoking US Women, National Health Interview Survey, 1997-2000

**CME Questions**

1. You are seeing a 48-year-old African American woman with newly diagnosed breast cancer. She has a history of hyperlipidemia and a body mass index (BMI) of 36 kg/m2. Based on the current study by Dr. Chang and colleagues, what can you tell this patient regarding the relationship between obesity and mortality among women with breast cancer?
  - There was no relationship between obesity and the relative risk of mortality
  - Only underweight women were at higher risk for mortality
  - Only overweight, but not obese, women were at higher risk for mortality
  - There was an increasing risk for mortality associated with increasing classes of obesity
2. How did the risk of mortality associated with obesity compare among women with and without breast cancer?
  - Obesity was associated with a higher risk of mortality only among women with breast cancer
  - Obesity was associated with a higher risk of mortality only among women without breast cancer
  - Obesity increased the risk of death to a greater degree among women with breast cancer compared with those without breast cancer
  - Obesity increased the risk of death to a greater degree among women without breast cancer compared with those with breast cancer
3. According to the current study, what role does this patient’s age play in the relationship between obesity and her risk of mortality?
  - Obesity was only associated with early mortality among women under 50 years old
  - Obesity was associated with the greatest number of life years lost among women under 50 years old
  - Obesity was only associated with early mortality among women aged 70 years or older
  - Obesity was associated with the greatest number of life years lost among women aged 70 years or older
4. According to the current study, what role does this patient’s race/ethnicity play in the relationship between obesity and her risk of mortality?
  - Obesity resulted in a greater number of life years lost among black women vs white women
  - Obesity resulted in a greater number of life years lost among other racial/ethnic groups compared with black and white women
  - Obesity only increased the risk of mortality among black women
  - Race/ethnicity did not affect the risk of mortality associated with obesity

**Evaluation**
